# Outcomes of patients with multiple myeloma and 1q gain/amplification receiving autologous hematopoietic stem cell transplant: the MD Anderson cancer center experience

**DOI:** 10.1038/s41408-023-00973-w

**Published:** 2024-01-10

**Authors:** Oren Pasvolsky, Sassine Ghanem, Denái R. Milton, Mikael Rauf, Mark R. Tanner, Qaiser Bashir, Samer Srour, Neeraj Saini, Paul Lin, Jeremy Ramdial, Yago Nieto, Guilin Tang, Yosra Aljawai, Hina N. Khan, Partow Kebriaei, Hans C. Lee, Krina K. Patel, Sheeba K. Thomas, Donna M. Weber, Robert Z. Orlowski, Elizabeth J. Shpall, Richard E. Champlin, Muzaffar H. Qazilbash

**Affiliations:** 1 Department of Lymphoma and Myeloma, The University of Texas MD Anderson Cancer Center, Houston, TX USA; 2 Lifespan Cancer Institute, Providence, RI USA; 3 Department of Medicine, Warren Alpert Medical School of Brown University, Providence, RI USA; 4 Department of Biostatistics, The University of Texas MD Anderson Cancer Center, Houston, TX USA; 5 Department of Stem Cell Transplantation and Cellular Therapy, The University of Texas MD Anderson Cancer Center, Houston, TX USA; 6 Department of Hematopathology, The University of Texas MD Anderson Cancer Center, Houston, TX USA; 7 Department of Hematology/Oncology, McGovern Medical School, The University of Texas Health Sciences Center at Houston, Houston, TX USA

**Keywords:** Myeloma, Risk factors

## Abstract

The prognostic impact of additional copies of chromosome 1q (1q + ) on outcomes of newly-diagnosed multiple myeloma (NDMM) patients undergoing autologous transplantation (autoSCT) is unclear. We conducted a retrospective single-center analysis of NDMM patients with 1q21 gain/amplification (3 or ≥4 copies of 1q, respectively) that received autoSCT between 2008–2018. 213 patients were included (79% 1q gain; 21% 1q amplification). The most commonly used induction regimen was bortezomib, lenalidomide, and dexamethasone (41%). At day100 post-autoSCT and at best post-transplant response, 78% and 87% of patients achieved ≥VGPR, and 38% and 50% achieved MRD-negative ≥VGPR, respectively. Median PFS and OS for the entire cohort were 35.5 months and 81.4 months, respectively. On multivariable assessment for PFS, MRD negative ≥VGPR before autoSCT (HR 0.52, p = 0.013) was associated with superior PFS, whereas 1q amplification was associated with inferior PFS (2.03, p = 0.003). On multivariate analysis for OS, achieving MRD negative ≥VGPR at best post-transplant response was associated with superior survival (0.29, p < 0.001), whereas R-ISS III and concomitant del17p or t(4:14) were associated with inferior survival (6.95, p = 0.030, 2.33, p = 0.023 and 3.00, p = 0.047, respectively). In conclusion, patients with 1q+ NDMM, especially 1q amplification, have inferior survival outcomes compared to standard-risk disease after upfront autoSCT, though outcomes are better than other high-risk cytogenetic abnormalities.

## Introduction

Additional copies of the long arm of chromosome 1 (1q + ) are among the most frequent cytogenetic abnormalities in patients with newly diagnosed multiple myeloma (NDMM), occurring in 20 to 50% of cases [1]. The frequency of 1q+ increases as the disease progresses from smoldering to relapsed/refractory disease [1, 2]. In MM cells harboring 1q + , upregulation of several genes at the1q locus, such as cyclin-dependent kinases regulatory subunit 1B (*CKS1B*), PDZ domain-containing scaffolding protein 1 (*PDZK1*), 26 S proteasome non-ATPase subunit 4 (*PSMD4*), Acidic Nuclear Phosphoprotein 32 Family Member E (*ANP32E*), interleukin enhancer binding factor 2 (*ILF-2*) and myeloid cell leukemia sequence 1 (*MCL-1*) have been postulated to contribute to oncogenesis and drive drug resistance [1, 3–6]. A higher proportion of patients with 1q+ are non-Caucasian, have IgA subtype disease, and have extramedullary MM involvement [7–10]. 1q+ has been further categorized into either 1q gain (3 copies) or 1q amplification ($\ge$4 copies). Some studies have suggested a worse prognosis for patients with amplification compared to those with gain, though there have been conflicting reports on this distinction [11–13]. The adverse prognostic impact of 1q+ has persisted in the era of novel therapy, including immunomodulatory drugs (IMIDs) and proteasome inhibitors (PIs), as evidenced by a meta-analysis of the National Cancer Research Institute Myeloma XI and Medical Research Council Myeloma IX trials, totaling 1905 NDMM patients, which showed that 1q+ was associated with a 68% increased risk of death [14].

Consolidation using high dose therapy with autologous stem cell transplant (autoSCT) is the current standard treatment for patients with MM, including those with high-risk cytogenetics [15]. Several studies have evaluated the outcomes of MM patients with 1q+ who received an autoSCT and have shown conflicting results. A study from the Mayo Clinic that included 155 1q+ NDMM patients who underwent upfront autoSCT, showed a shorter time to next treatment (TTNT) and overall survival (OS) in patients with 1q+ compared to patients without this abnormality, although there was no difference in outcomes between 1q gain and 1q amplification [13]. In contrast, in a subgroup analysis of the FORTE trial that compared induction with either carfilzomib, lenalidomide (Len) and dexamethasone (KRD) or carfilzomib, cyclophosphamide and dexamethasone (KCD) followed by autoSCT in NDMM, patients with 1q amplification had lower median progression-free survival (PFS) compared to patients with 1q gain or no 1q abnormality (21.8 months, 53 months and not reached, respectively) [11]. A summary of study design, patient characteristics and outcomes of selected previous reports on patients with 1q+ MM are presented in Supplementary Table 1.

Therefore, we decided to examine the real-world outcomes of patients with 1q+ NDMM, who received induction with contemporary anti-myeloma agents, upfront autoSCT and post-transplant maintenance at our institution.

## Methods

### Study design and participants

We conducted a retrospective, single-center, chart review study of patients with NDMM with additional copies of 1q who received autoSCT between 2008–2018. Data was obtained from our institution’s transplant database and chart-based review. The primary endpoints were PFS and OS, and the secondary endpoints were hematological response and minimal residual disease (MRD) status after autoSCT. The study was conducted after approval by the University of Texas MD Anderson Institutional Review Board (IRB) and in accordance with the Declaration of Helsinki and the 1996 Health Insurance Portability and Accountability Act (HIPAA).

### Response definitions and MRD evaluation

The International Myeloma Working Group (IMWG) criteria were used to evaluate the response, as well as progression [16]. Patients were categorized as having complete response (CR), stringent CR (sCR), very good partial response (VGPR), partial response (PR), stable disease (SD), or progressive disease (PD).

We also evaluated the MRD status by 8-color next-generation flow cytometry (NGF) at our institution. The sensitivity of our assay is 1/10^-5^ cells (0.001%) based on acquisition and analysis of at least 2 million events.

### Fluorescence in situ hybridization analysis

Fluorescence in situ hybridization (FISH) analysis was performed for the detection of known high-risk cytogenetic alterations, namely t(4;14), t(14;16), del(17p), and 1q21 gain or amplification by using the following FISH probe sets: IGH::FGFR3 dual-color dual-fusion probes; IGH::MAF dual-color dual-fusion probes; TP53/CEP17 dual-color and CDKN2C/CKS1B dual-color probes. 1q21 gain was defined as having 3 copies of CKS1B, while 1q21 amplification was identified as having ≥4 copies of CKS1B. Plasma cell enrichment was not routinely performed during the study period. The cut-off value for common abnormal signal patterns was established by our clinical cytogenetics laboratory as follows: 7.9% for 1q21 gain, 0% for 1q21 amplification, 4.7% for deletion of *TP53*, 0.4% for t(4;14), and 0% for t(14;16).

### Statistical methods

Patient, clinical characteristics, and response for all patients were summarized using descriptive statistics. PFS time was computed from the date of autoSCT to the date of disease progression or death (if died without disease progression) or the last follow-up date. Patients who were alive and did not experience progression of disease at the last follow-up date were censored. OS time was computed from the date of autoSCT to the last known vital sign. Patients alive at the last follow-up date were censored. PFS and OS were estimated using the Kaplan-Meier method and differences between groups were evaluated by the log-rank test. Associations between PFS and OS and measures of interest were determined using Cox proportional hazards regression models. Measures that occur after autoSCT (i.e., response and MRD after autoSCT as well as maintenance treatment) were included in the regression models as time-dependent covariates.

Statistical analyses were performed using SAS 9.4 for Windows (Copyright © 2002–2012 by SAS Institute Inc., Cary, NC). All statistical tests used a significance level of 5%. No adjustments for multiple testing were made.

#### Data sharing statement

The data that support the findings of this study are available on request from the corresponding author.

## Results

### Patient, disease, and treatment characteristics

Our analysis included a total of 213 NDMM patients with 1q abnormalities who underwent autoSCT at our institution. Median age was 62.5 (range 34–80) years and 53% (n = 113) were male. The majority of patients (n = 114, 54%) had Revised International Staging System (R-ISS) stage II disease while 40 (19%) and 22 (10%) patients had R-ISS stages I and III, respectively. The most commonly used induction regimen was bortezomib, Len and dexamethasone (VRD) (n = 88, 41%) and the conditioning regimen was most commonly melphalan (n = 165, 77%). Overall, 169 (79%) patients had one additional copy of 1q + , while 18 (8%) and 26 (12%) patients had 2 or >2 additional copies of 1q + , respectively. Thirty-one (15%) patients had concomitant del(17p), 30 (14%) had t(4;14), and 13 (6%) patients had t(14;16) as additional high-risk cytogenetic abnormalities. One-hundred and eighty-two (85%) patients received post-transplant maintenance, mostly with Len alone or with dexamethasone (58%). Patient characteristics for the entire cohort and by the number of copies of 1q are presented in Table 1. Compared to patients with 3 copies of 1q, those with >3 copies were more often female (43% vs. 61%, p = 0.041) and more often had higher stage of disease by R-ISS (p = 0.032) and by R2-ISS (p = 0.024).

**Table 1:** Patient characteristics.

		1q Copies		
Measure	All (N = 213)	3 (N = 169)	> 3 (N = 44)	p-value
**Gender, n (%)**				
Male	113 (53)	96 (57)	17 (39)	0.041
Female	100 (47)	73 (43)	27 (61)	
**Age at autoSCT (years)**				
Median (range)	62.5 (34.1–79.9)	61.8 (34.1–79.9)	64.3 (45.1–76.4)	0.12
**Race, n (%)**				
White	143 (68)	110 (66)	33 (77)	0.27
Black	41 (20)	34 (20)	7 (16)	
Hispanic	18 (9)	17 (10)	1 (2)	
Asian	7 (3)	5 (3)	2 (5)	
Unknown	4	3	1	
**Year of autoSCT, n (%)**				
2010–2014	59 (28)	44 (26)	15 (34)	0.34
2015–2018	154 (72)	125 (74)	29 (66)	
**R-ISS, n (%)**				
I	40 (23)	37 (26)	3 (8)	0.032
II	114 (65)	88 (63)	26 (72)	
III	22 (13)	15 (11)	7 (19)	
Unknown	37	29	8	
**R2-ISS, n (%)**				
II	39 (25)	36 (30)	3 (9)	0.024
III	88 (57)	68 (56)	20 (63)	
IV	27 (18)	18 (15)	9 (28)	
Unknown	59	47	12	
**ISS, n (%)**				
I	71 (38)	61 (41)	10 (26)	0.19
II	64 (34)	47 (31)	17 (44)	
III	54 (29)	42 (28)	12 (31)	
Unknown	24	19	5	
**Induction treatment, n (%)**				
KRD	44 (21)	34 (20)	10 (23)	0.87
VCD	27 (13)	23 (14)	4 (9)	
VD	29 (14)	24 (14)	5 (12)	
VRD	88 (42)	70 (42)	18 (42)	
Other	23 (11)	17 (10)	6 (14)	
Unknown	2	1	1	
**Conditioning regimen, n (%)**				
Bu/Mel based	42 (20)	34 (20)	8 (18)	0.60
Mel	165 (77)	129 (76)	36 (82)	
Other	6 (3)	6 (4)	0	
**Response prior to autoSCT, n (%)**				
sCR/CR	38 (18)	27 (16)	11 (25)	0.46
nCR/VGPR	98 (46)	78 (46)	20 (45)	
PR	62 (29)	52 (31)	10 (23)	
SD	6 (3)	4 (2)	2 (5)	
PD	9 (4)	8 (5)	1 (2)	
**MRD status prior to autoSCT, n (%)**				
Negative	82 (38)	64 (38)	18 (41)	0.89
Positive	126 (59)	101 (60)	25 (57)	
Not done	5 (2)	4 (2)	1 (2)	
**Presence of other high-risk cytogenetics, n (%)**				
del17	31 (15)	21 (12)	10 (23)	0.17
t(4;14)	30 (14)	24 (14)	6 (14)	0.97
t(14;16)	13 (6)	9 (5)	4 (9)	0.31
**Proportion of cells with 1q** + **(1 additional copy)**				
Number of patients	148	148		
Median (range)	0.2 (0.0–1.0)	0.2 (0.0–1.0)		
**Proportion of cells with 1q** + **(2 additional copies)**				
Number of patients	17		17	
Median (range)	0.2 (0.0–0.9)		0.2 (0.0–0.9)	
**Proportion of cells with 1q** + **(≥ 3 additional copies)**				
Number of patients	22		22	
Median (range)	0.3 (0.0–0.9)		0.3 (0.0–0.9)	
**Any post-transplant maintenance**				
No	31 (15)	27 (16)	4 (9)	0.34
Yes	182 (85)	142 (84)	40 (91)	
**Maintenance therapy, n (%)**				
Len +/- Dex	105 (58)	84 (59)	21 (53)	0.58
Len + PI +/- Dex	29 (16)	20 (14)	9 (23)	
Len/Elotuzumab	21 (12)	18 (13)	3 (8)	
Single agent PI	23 (13)	17 (12)	6 (15)	
Other	4 (2)	3 (2)	1 (3)	

*autoSCT* autologous hematopoietic stem cell transplant, *Bu/Mel* busulfan, melphalan, *CR* complete response, *Dex* dexamethasone, *ISS* International Staging System, *KRD* carfilzomib, lenalidomide, dexamethasone, *Len* lenalidomide *Mel* melphalan, *MRD* minimal residual disease, *n* number, *NA* not available, *nCR* near complete response, *PD* progressive disease, *PI* proteasome inhibitor, *PR* partial response, *R-ISS* Revised International Staging System, *sCR* stringent complete response, *SD* stable disease, *VCD* bortezomib, cyclophosphamide, dexamethasone, *VD* bortezomib, dexamethasone, *VGPR* very good partial response, *VRD* bortezomib, lenalidomide, dexamethasone.

### Responses and MRD outcomes

**Fig. 1:**
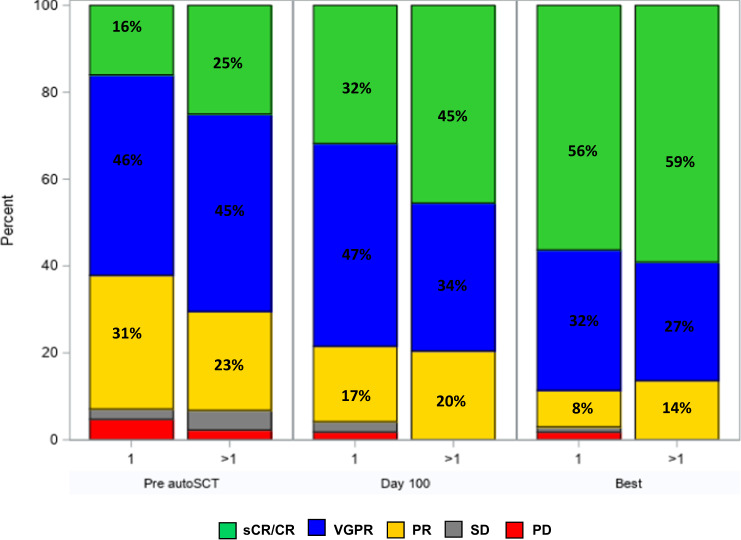
Pre-transplant, day 100 post-transplant and best post-transplant responses in patients with (1) or (>1) additional copies of 1q.

After completing induction therapy and prior to transplantation, 38 (18%) and 136 (64%) patients achieved ≥CR or ≥VGPR, respectively, and 69 (32%) achieved MRD negative ≥VGPR. Seventy-eight percent and 87% of patients had ≥VGPR at day 100 and at best response post-autoSCT, respectively. Thirty-eight percent and 50% of patients had an MRD negative ≥VGPR at day 100 and at best response post-autoSCT, respectively. Pre- and post-transplant hematological responses according to the number of additional copies of 1q+ are depicted in Fig. 1.

### Survival outcomes

**Fig. 2:**
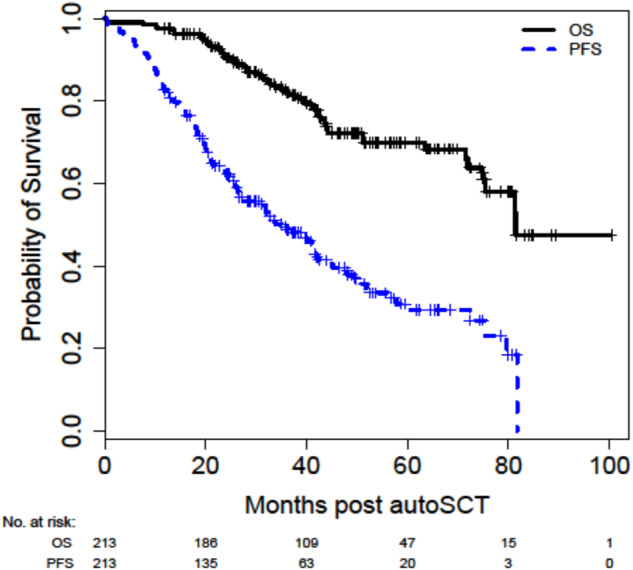
Overall and progression-free survival of patients with additional copies of 1q.

The median follow-up for the entire cohort was 41.1 (range 0.3–100.4) months, and for survivors was 43.9 (range 11.9–100.4) months. For the entire cohort, the median PFS and OS were 35.5 (95% CI 26.6–42.1) months and 81.4 (95% CI 74.9-not reached) months, respectively (Fig. 2).

**Fig. 3:**
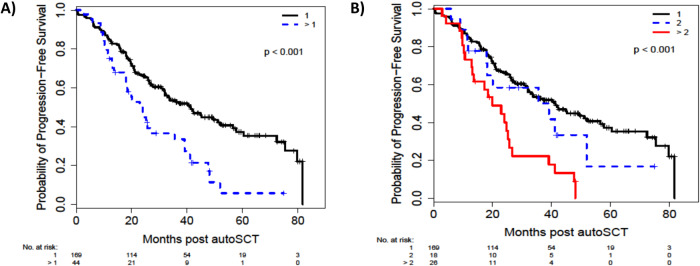
Progression-free survivalin patient subsets. **A** Progression-free survival in patients with (1) or (>1) additional copies of 1q. **B** Progression-free survival according to number of additional copies of 1q+ : (1) vs (2) vs (>2).

On univariable assessment (UVA) for PFS, achieving MRD negative ≥VGPR status prior to autoSCT (hazard ratio [95% CI], 0.49 [0.32–0.74]; p < 0.001) and at day 100 after autoSCT (0.59 [0.40–0.86]; p = 0.007) were associated with better PFS. Post-transplant maintenance with Len was also associated with improved PFS (0.56 [0.38–0.82]; p = 0.003) (Supplementary Table 2). Presence of >1 additional copies of 1q+ was associated with a worse PFS (2.03 [1.36–3.03]; p < 0.001, Fig. 3A), which was even worse in patients with >2 additional copies of 1q+ (2.66 [1.67–4.24]; p < 0.001, Fig. 3B). On multivariable assessment (MVA) for PFS, MRD negative ≥VGPR before autoSCT (0.52 [0.31–0.87]; p = 0.013) was associated with better outcomes, whereas 1q amplification was associated with worse PFS (2.03 [1.27–3.22]; p = 0.003) (Table 2).

**Table 2:** Summary of multivariable assessments for progression-free-survival.

Parameter	Hazard Ratio (95% CI)	p-value
**ISS**		
II vs I	1.47 (0.91, 2.36)	0.12
III vs I	0.91 (0.51, 1.63)	0.76
Unknown vs I	1.13 (0.54, 2.36)	0.75
**Prior MRD/Response**		
Negative/≥VGPR vs Other	0.52 (0.31, 0.87)	0.013
**Number of additional copies of 1q** +		
> 1 vs 1	2.03 (1.27, 3.22)	0.003
**t(4;14)**		
Present vs Absent	1.61 (0.77, 3.36)	0.20
Not done vs Absent	1.86 (0.93, 3.74)	0.08
**t(14;16)**		
Present vs Absent	2.26 (0.86, 5.91)	0.10
Not done vs Absent	0.69 (0.32, 1.52)	0.36
**100-day MRD/response**^**a**^		
Negative/≥VGPR vs Other	0.71 (0.46, 1.09)	0.12
**Maintenance therapy**^**a**^		
Lenalidomide vs Other	0.68 (0.45, 1.03)	0.07

*CI* Confidence interval, *ISS* International Staging System, *MRD* Minimal residual disease, *ref* reference group, *VGPR* very good partial response.

^a^Included in the model as a time-dependent covariate.

On UVA for OS, MRD negative ≥VGPR status prior to autoSCT (0.51 [0.26–0.99]; p = 0.046), at day 100 after autoSCT (0.43 [0.23–0.78]; p = 0.006) and at best post-transplant response (0.49 [0.28–0.86; p = 0.013), as well as use of Len-based post-transplant maintenance (0.45 [0.23–0.88]; p = 0.019) were all associated with better OS, while a higher R-ISS stage (stage II, 3.79 [1.15–12.42]; p = 0.028 and stage III, 5.95 [1.60–22.13]; p = 0.008, compared to stage I), presence of >1 additional copies of 1q+ (1.90 [1.07–3.37]; p = 0.028) and the presence of concomitant del17p (2.19 [1.17–4.11]; p = 0.014) were associated with worse OS (Supplementary Table 3). On MVA for OS, achieving MRD negative ≥VGPR at best post-transplant response was associated with better survival (0.29 [0.15–0.56; p < 0.001), whereas R-ISS stage III was associated with worse OS (6.95 [1.21–39.90; p = 0.030, compared with stage I) (Table 3).

**Table 3:** Summary of multivariable assessments for overall survival.

Parameter	Hazard Ratio (95% CI)	p-value
**R-ISS**		
II vs I	3.71 (0.85, 16.13)	0.08
III vs I	6.95 (1.21, 39.90)	0.030
Unknown vs I	4.53 (0.89, 23.10)	0.07
**Prior MRD/Response**		
Negative/≥VGPR vs. Other	0.43 (0.18, 1.04)	0.06
**Del17**		
Present vs Absent	2.33 (1.13, 4.82)	0.023
Not done vs Absent	0.45 (0.06, 3.58)	0.45
**Number of additional copies of 1q** +		
> 1 vs 1	1.88 (0.93, 3.83)	0.08
**t(4;14)**		
Present vs Absent	3.00 (1.02, 8.87)	0.047
Not done vs Absent	2.16 (0.73, 6.41)	0.16
**t(14;16)**		
Present vs Absent	2.82 (0.80, 9.97)	0.11
Not done vs Absent	0.47 (0.14, 1.54)	0.21
**Best MRD/response**^**a**^		
Negative/≥VGPR vs. Other	0.29 (0.15, 0.56)	< 0.001
**Maintenance therapy**^**a**^		
Lenalidomde vs. Other	0.67 (0.33, 1.36)	0.27

*CI* confidence interval, *MRD* Minimal residual disease, *ref* reference group, *R-ISS* Revised International Staging System, *VGPR* very good partial response.

^a^Included in the model as a time-dependent covariate.

Notably, the percentage of cells with 1q+ was not associated with PFS or OS, both when evaluated as a continuous variable, or as a categorical variable using thresholds of either 30% or 50% (Supplementary Table 2 and Supplementary Table 3).

### Impact of co-occurring cytogenetic abnormalities

The presence of concomitant del17p was associated with worse OS in UVA (2.19 [1.17–4.11]; p = 0.014) and in MVA (2.33 [1.13–4.82]; p = 0.023). Concomitant t (4:14) was not associated with worse OS in UVA (1.56 [0.71–3.46]; p = 0.27), yet was predictive of worse OS in MVA (3.00 [1.02–8.87]; p = 0.047, Table 3). None of the other high-risk cytogenetic abnormalities were predictive of PFS.

## Discussion

To the best of our knowledge, this is the largest contemporary cohort of 1q+ NDMM patients who received induction therapy followed by upfront autoSCT. We observed a median PFS of 35.5 months and a median OS of 81.4 months for the entire cohort. The presence of two or more additional copies of 1q+ was associated with inferior PFS in MVA, whereas achieving deeper responses before autoSCT was associated with improved PFS. Concomitant occurrence of del17p or t(4:14) was associated with worse OS in MVA.

Survival outcomes in our cohort were better than previous studies of outcomes in high-risk MM (HRMM) patients receiving upfront autoSCT. A previous report by our group found that HRMM patients had a median PFS and OS of 25 and 70 months, respectively, which are lower than the PFS and OS observed in this study [17]. Shah et al retrospectively reviewed patients with high-risk cytogenetics at Memorial Sloan Kettering Cancer Center who underwent treatment with novel agents and consolidation with autoSCT. Ninety-five patients were identified, of which 21% had 1q + . Among the patients with 1q + , the median PFS from diagnosis was 2.1 years and the median OS from diagnosis was 4.4 years, both of which are lower than those observed in the present study [9]. Similarly, a Center for International Blood and Marrow Transplant Research (CIBMTR) report on patients with HRMM who underwent autoSCT, showed a median PFS and OS of 21 and 68 months, respectively. However, in the subset of patients in the CIBMTR analysis with chromosome 1 abnormalities (1q+ or 1p-; n = 25), survival outcomes were comparable to those of patients with standard-risk MM, with a 3-year PFS of 50% and 3-year OS of 91% [18]. Furthermore, the survival outcomes for the 1q+ cohort were better than what we observed for other high-risk abnormalities at our institution. We observed median PFS and OS of 22.9 months and 60.4 months, respectively, in a cohort of 79 patients with t(4;14) who underwent upfront autoSCT [19]. Similarly, in a cohort of 79 patients with two or more high-risk abnormalities, we observed median PFS and OS of 22.9 months and 71.5 months, respectively [20].

A report from the Mayo Clinic evaluated the characteristics and outcomes of 391 NDMM patients with 1q+ diagnosed between 2006 and 2018, 155 of whom received frontline autoSCT. Compared to patients without 1q + , those with 1q+ had an inferior time to next treatment (TTNT) of 19.9 vs 27.7 months (p < 0.001) and increased risk of death (risk ratio [RR], 1.9; p < 0.001). In patients who underwent first line consolidation with autoSCT, TTNT was shorter in patients with 1q+ compared to those without 1q + , with a median TTNT of 29.8 vs 37.1 months (p = 0.01). Similarly, OS in patients who underwent autoSCT, whether frontline or later in their disease, was shorter in patients with 1q+ at 5.5 years compared to patients without 1q+ at 8.9 years (p < 0.001). The better survival outcomes in our cohort likely reflect differences in study populations, as only 42% of patients in that study underwent upfront transplant [13]. Furthermore, 36% of the 1q+ cohort in the study by the Mayo Clinic received induction with a PI and an IMID, compared to 62% of patients in the present study.

One of the important findings in our study is that patients with more than one additional copy of 1q + , i.e. 1q+ amplification, had worse PFS than those with 1q gain, despite autoSCT. Some previous studies have shown that the presence of ≥2 additional copies of 1q+ carries a worse prognosis [7, 21]. In a subgroup analysis of the FORTE trial that examined the impact of carfilzomib-based induction (KRD or KCD) followed by autoSCT in 400 NDMM patients with 1q data, PFS was progressively worse with an increasing 1q copy number, as median PFS was not reached in the normal 1q group (n = 219), while median PFS was 53 months in the 1q gain group (n = 129) and 21.8 months in the 1q amplification group (n = 52). The authors also observed a difference in OS when comparing patients with 1q amplification to those with 1q gain or without 1q+ (p < 0.001). In the current study we did not observe a difference in OS between patients with 1q gain compared to those with 1q amplification. In that study, subgroup analysis showed that KRD induction followed by autoSCT was able to abrogate the negative impact of 1q gain on OS (p = 0.565). However 1q amplification was still associated with inferior survival (p < 0.001) [11]. On the other hand, in the report from Mayo Clinic, there was no difference in outcomes between patients with 1q gain and those with 1q amplification: TTNT of 19.6 vs 14.4 months (p = 0.10) and OS of 4.9 vs 4.3 years (p = 0.21), respectively [13]. Of note, the percentage of patients in each subgroup who underwent autoSCT in that report was not specified. Similarly, a recent single-center report on 695 1q+ NDMM patients from China did not find a difference between 1q gain or amplification in terms of PFS (p = 0.48) or OS (p = 0.21) [22]. Only 37 patients in that cohort (5.3%) received upfront autoSCT.

In the present study, patients with 1q+ MM and concomitant del17p or t(4:14) had inferior OS in MVA. However, none of the additional high-risk cytogenetic abnormalities were associated with worse PFS. These results are similar to those reported by the Mayo clinic, which showed that patients with 1q+ and an additional high-risk IgH translocation had worse OS (p < 0.001), without a difference in TTNT [13]. In another analysis that included 201 patients with NDMM who received lenalidomide, bortezomib, dexamethasone (RVD) induction, 46.7% of the cohort had 1q + . Co-occurrence of other high-risk cytogenetic abnormalities was associated with worse PFS : [t(4:14), HR 4.18, p = 0.008], [t(14:16), HR 2.80, p = 0.036] and [del17p, HR 2.52, p = 0.01]. Impact of concomitant high-risk abnormalities on OS was not reported [23].

A previous propensity-matched analysis by Varma et al. at our institution examined the outcomes of 85 patients with 1q+ and/or 1p deletion (1p-) who underwent consolidation with autoSCT between 2006 and 2015. Sixty-seven (79%), 4 (5%), and 14 (16%) patients had only 1q + , only 1p-, or both, respectively. The median PFS for patients with 1q gain was 32.1 months compared to 20.0 months with 1q amplification, with a trend towards statistical significance (hazard ratio [95% CI], 0.49 [0.23–1.05]; p = 0.06). Median OS had not been reached for either copy number group, and no significant association with OS was found (p = 0.84) [24]. Compared to the study by Varma et al., in the current analysis we focused only on patients with 1q + , included a larger and more recent cohort of patients and had a considerably longer follow-up period (41.1 months vs. 29.2 months).

We found no significant difference in survival outcomes for patients with a larger proportion of MM cells with 1q + , using cutoffs of 30% and 50%. This is consistent with findings from most previous reports that also evaluated the impact of the proportion of MM cells with 1q abnormalities. In the Mayo clinic study, there was no statistically significant impact of the proportion of MM cells with 1q gain on OS, using either a 20% cutoff (p = 0.10) or a 50% cutoff (p = 0.42). Similarly, the proportion of cells with 1q amplification did not significantly impact OS, with a cutoff of 20% (p = 0.66) [13]. In a small retrospective study (n = 34) that mostly included patients that did not undergo autoSCT (n = 28, 82%), 1q+ clone size with a cutoff of 20% did not have an impact on either PFS (p = 0.2) or OS (p = 0.8) [25]. However, a single-center analysis from China by Wang et al. did observe worse median PFS in 1q+ patients with a clone size of ≥29% compared to those with a clone size of <29% (26.5 months vs. not reached, p < 0.001) [22]. In another single-center report from China that included 96 patients with NDMM and 1q + , outcomes were reported for three groups stratified by clone size: < 5%, 5%–20%, and > 20% [26]. Patients in the >20% group had inferior 2-year PFS compared to those in the <5% group (37.5% vs. 65.2%, p < 0.001), yet there was no significant difference in the PFS between the patients in the >20% and the 5–20% groups (p = 0.581). Similarly, patients in the >20% group had inferior 3-year OS compared with those in the <5% group (59.6% vs. 83.4%, p = 0.012), but OS was comparable between the >20% and the 5–20% groups (p = 0.488).

We would like to emphasize that the FISH tests used for detecting cytogenetic abnormalities in multiple myeloma belong to “laboratory developed tests” (LDT). The cut-off values for each abnormal signal pattern are established during the validation of the FISH probe. As LDT, these tests allow individual laboratories to set their own cut-off values for what constitutes an abnormal result. Consequently, there is a slight variance in these cut-off values among different laboratories [13, 26]. Furthermore, in the context of research, some studies may utilize “clonal size” as a threshold, rather than a traditional cut-off value for abnormality detection [27, 28]. In these cases, the significance of clonal size often hinges on reaching a certain high percentage, which varies depending on the specific research parameters and objectives.

Incorporation of anti-CD38 antibodies as part of quadruplet induction regimens has led to deeper and more durable remissions. Recently, the final analysis of the randomized phase II GRIFFIN trial has been published. Patients with NDMM who received daratumumab-RVD induction had higher rates of stringent CR (67% vs. 48%, p = 0.0079), and higher rates of 4-year PFS (87.2% vs. 70.0%, p = 0.032) compared to patients who received RVD induction. Per study design, all patients were intended to receive upfront autoHCT, and post-transplant consolidation and maintenance that differed according to treatment arm [29]. In the single-arm phase 2 MASTER trial, dara-carfilzomib, lenalidomide, dexamethasone (dara-KRD) induction followed by autoHCT led to promising rates of MRD negativity and 3-year PFS (71% and 88%, respectively) [30]. Callander et al. presented analyses of the GRIFFIN and MASTER trials according to cytogenetic risk at the 2022 American Society of Hematology (ASH) annual meeting [31]. Patients with one high-risk cytogenetic abnormality (including 1q + ) who received dara-based quadruplet therapy achieved high rates of ≥CR, MRD negativity and 2-year PFS rates. In the setting of relapsed/refractory MM, analyses of two independent phase III trials have shown that the addition of isatuximab to either pomalidomide, dexamethasone (Pd) or carfilzomib, dexamethasone (Kd) improves PFS to the same extent in patients with or without 1q21 + , and also for either gain or amplification of 1q [27]. In the present study, patients did not receive anti-CD38 antibody-based induction, and future studies will reveal long-term outcomes of high-risk MM patients, including those with 1q + , who receive quadruplet dara-based induction regimens followed by autoHCT.

Our study has several limitations inherent to its retrospective nature, including variabilities in care and possible unknown confounders that were not accounted for, despite the use of multivariable analysis. There was also a selection bias limiting the patient population to those who were transplant-eligible, which precludes conclusions regarding outcomes of all patients with 1q + . Another limitation is the lack of routine enrichment for plasma cell to perform FISH analysis in this patient population.

In conclusion, our study reaffirms 1q+ as an adverse cytogenetic abnormality in patients with MM, with relatively poor survival outcomes, despite the use of novel agents, autoSCT and post-transplant maintenance. However, patients with 1q+ may have better outcomes compared to patients with other high-risk cytogenetic abnormalities. Our study demonstrated worse PFS for patients with 1q amplification compared to those with 1q gain. Recently developed CAR T and bispecific T cell engagers should be tested in patients with high-risk cytogenetics, including 1q+ and specifically 1q amplification, and these patients may benefit from incorporation of these agents earlier in their disease course [32–34].

### Supplementary information

Supplementary Table 1

Supplementary Table 2

Supplementary Table 3

## Data Availability

The datasets generated during and/or analyzed during the current study are available from the corresponding author on reasonable request.
