# Supplementary material for: Outcomes of patients with multiple myeloma and 1q gain/amplification receiving autologous hematopoietic stem cell transplant: the MD Anderson cancer center experience
Source: Blood Cancer J. 2024 Jan 10;14(1):4. doi: 10.1038/s41408-023-00973-w (PMC10781953; doi:10.1038/s41408-023-00973-w)
Supplement: Supplementary file 1 — Supplementary Table 1 [file 41408_2023_973_MOESM1_ESM.docx]

Supplementary Table 1. Selected previous publications on patients with 1q+ multiple myeloma

| **Study** | **Study design** | **Population studied** | **Induction** | **Upfront autoSCT** | **with 1q+**  **n (%)** | **1q gain /**  **1q AMP** | **Post-autoSCT Maintenance** | **PFS** |
| --- | --- | --- | --- | --- | --- | --- | --- | --- |
| Shah et al. (2016) | Retrospective, single-center (MMSK) | 95 patients with NDMM between 2009-2012 | LEN-based (25%), BOR-based (39%), VRD (35%) | 95 (100%) | 20 (21%) | 15 (75%) /  5 (25%) | 73 (77%) | Median PFS 2.1 years (95% CI 1.2- NR) for 1q+; 4.3 years (3.3-NR) without 1q+ |
| Abdallah et al. (2020) | Retrospective, single-center (Mayo Clinic) | 1376 patients with NDMM between 2005-2018 | PI-based (36%), IMID-based (35%), PI+IMID-based (28%) | 581 (44%) | 391 (28%) | 105 (68%) /  50 (32%)^a^ | NA | Median TTNT 19.9 months (95% CI 17.2-22.9) for 1q+; 27.7 months (25.3-30.3) without 1q+ |
| D'Agostino et al. (2020) | Randomized controlled trial, multi-center (Italy) | 474 transplant-eligible patients with NDMM between 2015-2017 | KRD/KCD^c^ | NA^c^ | 181 (45%)^b^ | 129 (71%) /  52 (29%) | NA^c^ | Median PFS 21.8 months for 1q AMP, 53 months for 1q gain, NR without 1q+ |
| Wang et al. (2022) | Retrospective, single-center (Zhongshan Hospital, China) | 781 patients with NDMM between 2013-2021 | PI-based (47%), IMID-based (3%), PI+IMID-based (51%) | 82 (12%) | 405 (52%) | 208 (65%) / 129 (38%) | NA | Median PFS 36.0 months (95% CI 28.0-NR) for 1q+, 71.1 months (71.1-NR) without 1q+ |

Abbreviations: AMP = amplification; autoSCT = autologous hematopoietic stem cell transplant; BOR = bortezomib; CI = confidence interval; IMID = immunomodulatory drug; KCD = carfilzomib, cyclophosphamide, dexamethasone; KRD = carfilzomib, lenalidomide, dexamethasone; LEN = lenalidomide; MMSK = Memorial Sloan Kettering Cancer Center; NA = not available; NDMM = newly-diagnosed multiple myeloma; NR = not reached; PI = proteosome inhibitor; PFS = progression-free survival; TTNT = time to next treatment; VRD = bortezomib, lenalidomide, dexamethasone; 1q+ = q1 gain/amplification; 1qNL = normal 1q.

^a^ of 155 patients with detailed FISH data

^b^ of 400 patients with available FISH data

^c^ per trial design, patients were randomly assigned [KRD+ up-front autoHCT]/[KRD]/[KCD+ up-front autoHCT] in a ratio of 1:1:1, followed by maintenance. Exact numbers for the 1q+ analysis not available
