# Supplementary material for: Outcomes of patients with multiple myeloma and 1q gain/amplification receiving autologous hematopoietic stem cell transplant: the MD Anderson cancer center experience
Source: Blood Cancer J. 2024 Jan 10;14(1):4. doi: 10.1038/s41408-023-00973-w (PMC10781953; doi:10.1038/s41408-023-00973-w)
Supplement: Supplementary file 2 — Supplementary Table 2 [file 41408_2023_973_MOESM2_ESM.docx]

Supplementary Table 2: Summary of Progression-Free-Survival: Univariable Assessments

| **Parameter** | **Hazard Ratio (95% CI)** | **p-value** |
| --- | --- | --- |
|  |  |  |
| **Age** | 1.01 (0.99, 1.03) | 0.25 |
| **Sex** |  |  |
| Female vs Male | 1.18 (0.83, 1.68) | 0.36 |
| **Year of autoSCT** |  |  |
| 2015-2018 vs 2010-2014 | 0.80 (0.55, 1.17) | 0.25 |
| **R-ISS** |  |  |
| II vs I | 1.42 (0.86, 2.35) | 0.17 |
| III vs I | 1.75 (0.89, 3.48) | 0.11 |
| Unknown vs I | 1.56 (0.86, 2.84) | 0.14 |
| **R2-ISS** |  |  |
| III vs II | 1.23 (0.73, 2.06) | 0.43 |
| IV vs II | 1.71 (0.88, 3.30) | 0.11 |
| Unknown vs II | 1.34 (0.78, 2.32) | 0.29 |
| **ISS** |  |  |
| II vs I | 1.82 (1.18, 2.81) | **0.007** |
| III vs I | 1.08 (0.66, 1.78) | 0.75 |
| Unknown vs I | 1.19 (0.64, 2.21) | 0.59 |
| **Induction regimens** |  |  |
| KRD vs VRD | 0.74 (0.44, 1.25) | 0.27 |
| VD vs VRD | 0.65 (0.37, 1.14) | 0.14 |
| VCD vs VRD | 0.82 (0.48, 1.39) | 0.46 |
| Other vs VRD | 0.90 (0.50, 1.62) | 0.72 |
| **Conditioning regimen** |  |  |
| Bu/Mel based vs Mel | 0.89 (0.55, 1.44) | 0.64 |
| Other vs Mel | 1.62 (0.59, 4.41) | 0.35 |
| **Hematologic response prior to transplant** |  |  |
| nCR/VGPR vs sCR/CR | 1.10 (0.64, 1.91) | 0.72 |
| PR vs sCR/CR | 1.51 (0.85, 2.66) | 0.16 |
| SD vs sCR/CR | 1.80 (0.60, 5.38) | 0.29 |
| PD vs sCR/CR | 6.79 (2.85, 16.17) | **< 0.001** |
| **MRD status prior to transplant** |  |  |
| Positive vs Negative | 1.77 (1.21, 2.60) | **0.003** |
| Not done vs Negative | 0.76 (0.18, 3.14) | 0.70 |
| **Prior MRD/response** |  |  |
| Negative/≥VGPR vs Other | 0.49 (0.32, 0.74) | **< 0.001** |
| **Del17p** |  |  |
| Present vs Absent | 1.17 (0.71, 1.94) | 0.54 |
| Not done vs Absent | 1.15 (0.53, 2.47) | 0.73 |
| **t(4;14)** |  |  |
| Present vs Absent | 1.55 (0.89, 2.68) | 0.12 |
| Not done vs Absent | 1.16 (0.78, 1.75) | 0.47 |
| **t(14;16)** |  |  |
| Present vs Absent | 1.01 (0.44, 2.32) | 0.98 |
| Not done vs Absent | 1.27 (0.83, 1.96) | 0.27 |
| **Number of additional copies of 1q+** |  |  |
| >1 vs 1 | 2.03 (1.36, 3.03) | **< 0.001** |
| 2 vs 1 | 1.37 (0.73, 2.57) | 0.32 |
| >2 vs 1 | 2.66 (1.67, 4.24) | **< 0.001** |
| **Proportion of cells with 1q+ (1 additional copy)** |  |  |
| continuous | 0.72 (0.28, 1.83) | 0.49 |
| >30% vs ≤ 30% | 0.77 (0.49, 1.22) | 0.27 |
| >50% vs ≤ 50% | 0.90 (0.52, 1.56) | 0.71 |
| **Proportion of cells with 1q+ (2 additional copies), continuous** | 0.56 (0.04, 7.51) | 0.66 |
| **Proportion of cells with 1q+ (≥ 3 additional copies), continuous** | 0.83 (0.13, 5.25) | 0.84 |
| **100-day response^a^** |  |  |
| CR vs non-CR | 0.59 (0.40, 0.88) | **0.009** |
| ≥VGPR vs <VGPR | 0.65 (0.43, 1.00) | **0.049** |
| **100-day MRD/response^a^** |  |  |
| Negative/≥VGPR vs Other | 0.59 (0.40, 0.86) | **0.007** |
| **Best MRD/response^a^** |  |  |
| Negative/≥VGPR vs Other | 0.76 (0.52, 1.12) | 0.17 |
| **Maintenance therapy^a^** |  |  |
| Yes vs No | 0.65 (0.41, 1.02) | 0.064 |
| Len vs non-Len | 0.56 (0.38, 0.82) | **0.003** |
| Len-based vs non-Len-based | 0.56 (0.35, 0.90) | **0.017** |

^a^ Included in the model as a time-dependent covariate.

**Abbreviations:** autoSCT = autologous hematopoietic stem cell transplant; Bu/Mel = busulfan, melphalan; CI = Confidence interval; CR = complete response; ISS = International Staging System; KRD = Carfilzomib, lenalidomide, dexamethasone; Len = Lenalidomide; Mel = melphalan; MRD = Minimal residual disease; nCR = near complete response; PD = progressive disease; PR = partial response; R-ISS = Revised international staging system; sCR = stringent complete response; SD = stable disease; VCD = bortezomib, cyclophosphamide, dexamethasone; VD = bortezomib, dexamethasone; VGPR = very good partial response; VRD = bortezomib, lenalidomide, dexamethasone.
