# Supplementary material for: Outcomes of patients with multiple myeloma and 1q gain/amplification receiving autologous hematopoietic stem cell transplant: the MD Anderson cancer center experience
Source: Blood Cancer J. 2024 Jan 10;14(1):4. doi: 10.1038/s41408-023-00973-w (PMC10781953; doi:10.1038/s41408-023-00973-w)
Supplement: Supplementary file 3 — Supplementary Table 3 [file 41408_2023_973_MOESM3_ESM.docx]

Supplementary Table 3: Summary of Overall Survival: Univariable Assessments

| **Parameter** | **Hazard Ratio (95% CI)** | **p-value** |
| --- | --- | --- |
|  |  |  |
| **Age** | 1.02 (0.99, 1.05) | 0.16 |
| **Sex** |  |  |
| Female vs Male | 1.54 (0.90, 2.62) | 0.11 |
| **Year of autoSCT** |  |  |
| 2015-2018 vs 2010-2014 | 0.72 (0.42, 1.26) | 0.25 |
| **R-ISS** |  |  |
| II vs I | 3.79 (1.15, 12.42) | **0.028** |
| III vs I | 5.95 (1.60, 22.13) | **0.008** |
| Unknown vs I | 4.80 (1.36, 17.00) | **0.015** |
| **R2-ISS** |  |  |
| III vs II | 1.86 (0.71, 4.93) | 0.21 |
| IV vs II | 2.85 (0.94, 8.60) | 0.06 |
| Unknown vs II | 2.47 (0.92, 6.64) | 0.07 |
| **ISS** |  |  |
| II vs I | 1.74 (0.86, 3.50) | 0.12 |
| III vs I | 1.36 (0.64, 2.87) | 0.42 |
| Unknown vs I | 1.71 (0.68, 4.32) | 0.26 |
| **Induction regimens** |  |  |
| KRD vs VRD | 0.44 (0.15, 1.26) | 0.13 |
| VD vs VRD | 0.88 (0.40, 1.94) | 0.75 |
| VCD vs VRD | 0.88 (0.40, 1.95) | 0.75 |
| Other vs VRD | 1.16 (0.52, 2.55) | 0.72 |
| **Conditioning regimen** |  |  |
| Bu/Mel based vs Mel | 0.79 (0.36, 1.76) | 0.57 |
| Other vs Mel | 2.14 (0.52, 8.91) | 0.29 |
| **Hematologic response prior to transplant** |  |  |
| nCR/VGPR vs sCR/CR | 0.85 (0.36, 2.02) | 0.72 |
| PR vs sCR/CR | 1.10 (0.45, 2.66) | 0.84 |
| SD vs sCR/CR | 2.68 (0.69, 10.44) | 0.15 |
| PD vs sCR/CR | 6.49 (2.26, 18.65) | **< 0.001** |
| **MRD status prior to transplant** |  |  |
| Positive vs Negative | 2.08 (1.13, 3.84) | **0.018** |
| Not done vs Negative | 1.52 (0.20, 11.61) | 0.69 |
| **Prior MRD/response** |  |  |
| Negative/≥VGPR vs Other | 0.51 (0.26, 0.99) | **0.046** |
| **Del17** |  |  |
| Present vs Absent | 2.19 (1.17, 4.11) | **0.014** |
| Not done vs Absent | 0.72 (0.17, 2.97) | 0.65 |
| **t(4;14)** |  |  |
| Present vs Absent | 1.56 (0.71, 3.46) | 0.27 |
| Not done vs Absent | 0.98 (0.53, 1.80) | 0.94 |
| **t(14;16)** |  |  |
| Present vs Absent | 2.01 (0.75, 5.41) | 0.17 |
| Not done vs Absent | 1.02 (0.53, 1.96) | 0.96 |
| **Number of additional copies of 1q+** |  |  |
| >1 vs 1 | 1.90 (1.07, 3.37) | **0.028** |
| 2 vs 1 | 2.06 (0.86, 4.89) | 0.10 |
| >2 vs 1 | 1.82 (0.93, 3.57) | 0.08 |
| **Proportion of cells with 1q+ (1 additional copy)** |  |  |
| continuous | 0.77 (0.19, 3.12) | 0.72 |
| >30% vs ≤ 30% | 0.93 (0.47, 1.85) | 0.84 |
| >50% vs ≤ 50% | 0.93 (0.41, 2.15) | 0.87 |
| **Proportion of cells with 1q+ (2 additional copies), continuous** | 2.02 (0.14, 28.07) | 0.60 |
| **Proportion of cells with 1q+ (≥ 3 additional copies), continuous** | 6.45 (0.68, 60.76) | 0.10 |
| **100-day response^a^** |  |  |
| CR vs non-CR | 0.56 (0.30, 1.05) | 0.07 |
| ≥VGPR vs <VGPR | 0.43 (0.24, 0.75) | **0.003** |
| **100-day MRD/response^a^** |  |  |
| Negative/≥VGPR vs Other | 0.43 (0.23, 0.78) | **0.006** |
| **Best MRD/response^a^** |  |  |
| Negative/≥VGPR vs Other | 0.49 (0.28, 0.86) | **0.013** |
| **Maintenance therapy^a^** |  |  |
| Yes vs No | 0.62 (0.32, 1.17) | 0.14 |
| Len vs non-Len | 0.58 (0.33, 1.05) | 0.07 |
| Len-based vs non-Len-based | 0.45 (0.23, 0.88) | **0.019** |

^a^ Included in the model as a time-dependent covariate.

**Abbreviations:** autoSCT = autologous hematopoietic stem cell transplant; Bu/Mel = busulfan, melphalan; CI = Confidence interval; CR = complete response; ISS = International Staging System; KRD = Carfilzomib, lenalidomide, dexamethasone; Len = Lenalidomide; Mel = melphalan; MRD = Minimal residual disease; nCR = near complete response; PD = progressive disease; PR = partial response; R-ISS = Revised international staging system; sCR = stringent complete response; SD = stable disease; VCD = bortezomib, cyclophosphamide, dexamethasone; VD = bortezomib, dexamethasone; VGPR = very good partial response; VRD = bortezomib, lenalidomide, dexamethasone.
